# Post-translational modifications in heat stress-related diseases

**DOI:** 10.3389/fmolb.2025.1666874

**Published:** 2025-09-22

**Authors:** Qi Cui, Keran Jia, Fang Li, Jie Zheng, Fukun Wang

**Affiliations:** 1 Clinical Laboratory, Bethune International Peace Hospital, Shijiazhuang, Hebei, China; 2 Nursing Department, Bethune International Peace Hospital, Shijiazhuang, Hebei, China

**Keywords:** heat stress, post-translational modifications, phosphorylation, acetylation, ubiquitination, methylation, S-nitrosylation

## Abstract

Post-translational modifications (PTMs) act as pivotal molecular hubs integrating heat stress signals into cellular responses driving heat-related diseases like heatstroke. This review synthesizes evidence demonstrating that dynamic PTM networks—including phosphorylation, acetylation, ubiquitination, methylation, SUMOylation, and S-nitrosylation—orchestrate pathophysiology through three distinctive mechanisms: PTM crosstalk, tissue-specific PTM signatures defining organ vulnerability, and translational utility. The potentials of PTM alterations as novel biomarkers for early diagnosis/prognosis and PTM-targeted interventions as therapeutic strategies are discussed. By delineating how PTMs reconfigure proteostasis, metabolism, and inflammation, this review provides a mechanistic framework for targeting PTM pathways to mitigate heatstroke and related conditions.

## 1 Introduction

Heat stress, the core physiological response of the body to high-temperature environments, exhibits dual effects: moderate heat stress elicits adaptive protective responses, while excessive heat exposure triggers pathological damage. Moderate heat stress can activate the conserved heat shock response (HSR), upregulate molecular chaperones to maintain protein homeostasis, and confer protective tolerance to cells (Singh et al., 2024). However, when heat exposure exceeds physiological limits, it drives pathological damage, inducing protein denaturation, oxidative stress, and systemic inflammation, ultimately causing heatstroke (HS)—a life-threatening condition with core body temperature >40 °C and multi-organ failure (Wang et al., 2025; Baindara et al., 2025). Beyond typical heatstroke, heat stress can exacerbate the pathological processes of cardiovascular events, neurodegenerative diseases, and chronic inflammatory diseases.

In this pathological network—encompassing heat stress-induced protein denaturation, oxidative stress, systemic inflammation, and exacerbated progression of cardiovascular, neurodegenerative, and inflammatory diseases—post-translational modifications (PTMs) act as precise regulators of protein functions. By covalently adding chemical groups such as phosphate, acetyl, and ubiquitin chains, PTMs dynamically control protein activity, localization, stability, and interaction networks, serving as molecular hubs linking heat stress stimuli to cell fate decisions. Recent studies have shown that heat stress can extensively reshape the modification profiles of PTMs such as phosphorylation, acetylation, ubiquitination, methylation, and S-nitrosylation, thereby regulating stress signal transduction, energy metabolism, and cell death programs, ultimately driving organ damage (Yuan et al., 2022; Durham et al., 2008) (Table 1). Therefore, this review focuses on how heat stress disrupts cellular homeostasis and promotes the development of diseases like heatstroke by reconstructing PTM networks. Can these PTM changes serve as novel biomarkers and therapeutic targets? By reviewing the research progress of major PTMs in heat stress-related diseases, particularly heatstroke, this review aims to reveal the pathophysiological significance of PTMs and provide a theoretical basis for in-depth analysis of pathogenesis and the development of targeted intervention strategies.

**TABLE 1:** Definitions and brief explanations of key PTMs discussed.

PTM type	Definition	Key regulatory enzymes	Major functions in heat stress-related diseases
Phosphorylation	Covalent addition of phosphate groups to serine, threonine, or tyrosine residues	Kinases (e.g., p38 MAPK, MK2, ERK); Phosphatases	Regulates HSP activation (e.g., HSP27), signal transduction (e.g., NF-κB pathway), and stress granule assembly
Acetylation	Addition of acetyl groups to lysine residues	Acetyltransferases; Deacetylases (e.g., HDACs)	Modulates mitochondrial enzyme activity (e.g., TCA cycle enzymes), histone structure, and HSP transcription
Ubiquitination	Covalent binding of ubiquitin to target proteins	E1 (activating), E2 (conjugating), E3 (ligating) enzymes (e.g., Nedd4, MDM2)	Mediates degradation of misfolded proteins (via UPS) and stress granule disassembly; regulates p53 stability
Methylation	Addition of methyl groups to lysine/arginine residues	Methyltransferases (e.g., PKMTs, PRMTs); Demethylases	Regulates histone modifications (e.g., H3K4me3) and transcriptional memory of heat adaptation genes
SUMOylation	Conjugation of SUMO proteins (SUMO1/2/3) to lysine residues	SUMOylases (e.g., Ubc9); DeSUMOylases	Enhances HSF1 transcriptional activity; modulates protein localization and DNA damage repair
S-nitrosylation	Formation of S-nitrosothiols via reaction of NO with cysteine thiols	Nitric oxide synthase (NOS); GSNOR (denitrosylase)	Alters redox enzyme activity (e.g., GSH-Px); triggers Ca^2+^leakage via RyR1 modification in heatstroke

## 2 Effects of heat stress on cellular protein homeostasis and PTM regulatory networks

Heat stress, as an intense physiological disturbance, impacts cellular protein homeostasis through multi-level mechanisms and profoundly reshapes the dynamic regulatory network of PTMs. When core body temperature rises abnormally, cells first activate the conserved HSR, whose core regulator, heat shock factor 1 (HSF1), is itself precisely controlled by PTMs. Phosphorylation (e.g., p38 MAPK-mediated Ser303/Ser307 sites) and acetylation modifications synergistically regulate HSF1 trimerization, nuclear translocation, and DNA-binding capacity, thereby driving the expression of molecular chaperones HSP70/HSP90 to maintain the folding and repair of damaged proteins (Shao et al., 2020; Brunet Simioni et al., 2009). However, when heat stress intensity exceeds the physiological compensation limit, leading to heatstroke, protein denaturation, misfolding, and aggregation are exacerbated, inducing endoplasmic reticulum stress and mitochondrial dysfunction (Roths et al., 2024; He et al., 2025). At this point, the ubiquitin-proteasome system (UPS) and autophagy pathways are activated to label and degrade abnormal proteins through ubiquitination (Comyn et al., 2014; Maxwell et al., 2021). Notably, heat stress-induced NADPH oxidase activation and mitochondrial reactive oxygen species (ROS) bursts further directly attack cysteine residues through S-nitrosylation, altering the functions of key metabolic enzymes and signaling proteins, forming a vicious cycle of protein homeostasis collapse (Qiao et al., 2022).

The cytoprotective roles of HSPs (e.g., HSP70, HSP90) and small heat shock proteins (sHSPs, e.g., HSP27) are not restricted to heat stress responses. They are widely involved in mitigating pathological processes in other diseases: for instance, HSP70 inhibits protein aggregation in neurodegenerative disorders such as Alzheimer’s and Parkinson’s disease (Lu et al., 2014); HSP27 modulates oxidative stress and inflammation in cardiovascular diseases, including myocardial infarction and atherosclerosis (Zou et al., 2023); and HSP90 regulates oncoprotein stability in various cancers, serving as a therapeutic target (Liang et al., 2024). These conserved functions—such as maintaining protein homeostasis, suppressing excessive inflammation, and inhibiting cell death—align with their roles in heatstroke, where they counteract protein denaturation, oxidative stress storms, and systemic inflammation. However, a detailed analysis of their roles in non-heat stress diseases is beyond the scope of this review, which focuses on their PTM-mediated regulation and functions in heat stress-related pathologies.

At the signal transduction level, heat stress rapidly activates core stress pathways such as MAPK, NF-κB, and AMPK (Li et al., 2024). The cascade reactions of these pathways are essentially dynamic PTM transmission processes. Elizabeth et al. found that high temperatures activate p38 MAPK through phosphorylation cascades, and its downstream target MK2 further phosphorylates Ser82/Ser78 sites of HSP27, promoting stress granule assembly and lysosomal autophagy to clear damaged organelles (Gallagher et al., 2024). Meanwhile, heat stress inhibits AMPK Thr172 phosphorylation in cardiomyocytes, disrupting glucose/lipid metabolism, reducing ATP production, and causing lactic acid accumulation (Tian et al., 2023). These modification events are not isolated but form complex “PTM crosstalk.” SUMOylation of HSF1 at K298 enhances its phosphorylation level, synergistically amplifying heat shock gene transcription (Brunet Simioni et al., 2009); while p53 stability is bidirectionally regulated by phosphorylation activation and MDM2-mediated ubiquitin degradation, determining cell fate under heat stress (Wang and Chen, 2003; Mansky et al., 2023).

Heat stress also indirectly disturbs PTM networks through metabolic reprogramming. Mitochondrial dysfunction reduces NAD^+^ levels, inhibiting class III deacetylase (Sirtuins) activity, leading to the accumulation of acetylated modifications in metabolic enzymes, which inhibits the tricarboxylic acid cycle and exacerbates energy crises (Martínez-Reyes and Chandel, 2020; Arnold and Finley, 2023). Additionally, heat stress induces histone modification remodeling such as H3K4me3 methylation and H3/H4 acetylation, regulating the transcriptional plasticity of heat adaptation-related genes through epigenetic memory mechanisms and affecting the body’s tolerance to repeated heat stimuli (Friedrich et al., 2021). In summary, heat stress globally disrupts PTM networks by directly modifying key proteins, restructuring PTM enzyme activity, and altering metabolic substrate levels. This process is both a core mechanism of cellular defensive responses and a driving hub of pathological damage.

## 3 Research on the correlation between heatstroke and PTMs

PTMs refer to processes that chemically modify amino acid residues of proteins after synthesis, altering protein structure and properties through covalent bonding of chemical groups or hydrolysis, thereby regulating protein functions, subcellular localization, stability, transcriptional activity, and interactions with other molecules. Common PTM types include phosphorylation, acetylation, ubiquitination, methylation, glycosylation, SUMOylation, and S-nitrosylation.

### 3.1 Changes and roles of phosphorylation modifications in heatstroke

As one of the most widely studied post-translational modifications, protein phosphorylation is catalyzed by protein kinases, which transfer phosphate groups from ATP to specific amino acid residues (e.g., serine, threonine, tyrosine) of proteins (Humphrey et al., 2015). The introduced phosphate groups can change protein conformation, activity, and interactions with other proteins or substrates. This modification is often reversible, with dephosphorylation catalyzed by protein phosphatases.

During heatstroke, the phosphorylation levels of numerous signal pathway-related proteins in cells change significantly (Sluzala et al., 2025; Sun et al., 2024). The MAPK pathway, a key cell signaling pathway, is significantly activated, with increased phosphorylation levels of key proteins ERK, JNK, and p38, which regulate the activity of downstream transcription factors, affect the expression of stress-related genes such as heat shock proteins, and participate in the regulation of cellular reactivity and tolerance to heat stress (Zhang and Liu, 2002). HSP27 (HSPB1), a member of the small heat shock protein family, exerts its functions mainly through phosphorylation. In cells exposed to drug toxicity, tumor proliferation, oxidative stress, and heat stress, the phosphorylation sites and aggregation forms of HSP27 vary. Gallagher et al. found that in response to heat stress-induced lysosomal damage, activated p38 MAPK/MK2 phosphorylates Ser135 and Arg136 residues of HSP27 after heat stress. Phosphorylated HSP27 recruits to stressed lysosomes and interacts with p62 bodies on the lysosomal surface, promoting lysosomal autophagy (Gallagher et al., 2024), which is crucial for clearing damaged lysosomes and maintaining cell viability. Although HSP27 is the primary sHSP implicated in heatstroke, other sHSPs like HSPB4 and HSPB5 exhibit protective roles in oxidative stress and proteinopathies (Sluzala et al., 2025; Phadte et al., 2021). Their direct involvement in heat stress remains underexplored and warrants future study. Notably, phosphorylation at specific residues of HSP27 is critical for its functional engagement in heat stress responses. However, it is important to distinguish between these mutant-based approaches and studies of *bona fide* phosphorylated HSP27: the latter reflects dynamic regulation by upstream kinases in response to heat stress, whereas mutations lock the protein in a static state, potentially overriding physiological feedback loops.

Beyond HSP27, MAPK-mediated phosphorylation also modulates inflammatory pathways, particularly through NF-κB regulation. In high-temperature environments, MAPK/ERK phosphorylates IκBα at Ser74, promoting NF-κB nuclear translocation to activate cell survival-related genes against heat stress and regulate oyster adaptation to environmental temperature changes (Wang et al., 2024). Consistently, Liu et al. found that exposure to high temperatures increases the phosphorylation of NF-κB p65 and IκBα, promoting the expression of the anti-apoptotic protein HSP27 to resist early apoptosis of human umbilical vein endothelial cells (HUVECs) induced by heat stress and maintain body homeostasis (Liu et al., 2015). However, a study on bone marrow macrophages suggested that during heatstroke, systemic inflammatory responses occur, and phosphorylated NF-κB translocates into the nucleus, activating inflammation-related genes and inducing the production of large amounts of pro-inflammatory cytokines such as TNF-α and IL-1β, thereby exacerbating heatstroke-related inflammatory responses and tissue damage (Cooper et al., 2010). Małgorzata et al. quantitatively analyzed on heat shock-induced attenuation of NF-κB signaling in human osteosarcoma cells using real-time single-cell imaging and mathematical modeling, showing that exposure to high temperatures inhibits the function of the NF-κB signaling pathway. The ability of NF-κB p65 to sense TNF-α is weakened, involving mechanisms such as high-temperature-induced IKK denaturation, inhibition of IκB degradation, NF-κB phosphorylation, and nuclear translocation, thereby suppressing the expression of its downstream target genes (Kardyńska et al., 2018; Paszek et al., 2020). In conclusion, high temperatures regulate NF-κB responses in single cells in complex and non-intuitive ways, which need to be considered in hyperthermia-based clinical treatment strategies.

Additionally, a study on the mechanism of organ damage induced by hyperthermia showed that heat stress enhances Z-DNA binding protein 1 (ZBP1) expression by inducing the binding of HSF1 to the promoter of ZBP1 gene, promoting RIPK3 kinase to phosphorylate its substrate mixed lineage kinase domain-like protein (MLKL), inducing various programmed cell deaths, and leading to disseminated intravascular coagulation and multiple organ damage (Yuan et al., 2022). A study on exertional heat stroke (EHS) in mice showed that HS induces delayed glucose and lipid metabolism dysfunction in cardiomyocytes, characterized by glycolytic reprogramming, accumulation of free fatty acids, and impaired tricarboxylic acid cycle (Laitano et al., 2020). Under normal circumstances, AMPK phosphorylation (e.g., at Thr172) is known to promote glucose uptake and fatty acid oxidation to increase ATP production (Rodríguez et al., 2021); in the context of heat stress, AMPK inhibition is associated with enhanced glycolysis, lactic acid accumulation, increased fatty acid synthesis, and reduced β-oxidation, leading to energy interruption, cell death, and cardiac dysfunction. Therefore, promoting AMPK Thr172 phosphorylation may be a potential strategy to alleviate HS-induced myocardial damage, based on its known roles in regulating glucose and lipid metabolism (Tian et al., 2023; Zheng et al., 1985; Roths et al., 2023).

### 3.2 Changes and roles of acetylation modifications in heatstroke

Acetylation modifications typically occur on lysine residues of proteins, with acetyltransferases transferring acetyl groups from acetyl-CoA to target proteins, affecting protein stability, DNA-binding capacity, and subcellular localization (Shang et al., 2022). Reversible regulation by deacetylases is also involved.

Studies have shown that during heatstroke, the acetylation levels of some metabolic enzymes in mitochondria change, affecting mitochondrial energy metabolism (Fang et al., 2024). Acetylation of key enzymes involved in the tricarboxylic acid cycle inhibits their activity, reducing cellular energy production and exacerbating cellular dysfunction induced by heat stress (Nunes-Nesi et al., 2013; Xiong and Guan, 2012). Pyruvate dehydrogenase (PDH), a key enzyme in cellular metabolism involved in the oxidative decarboxylation of pyruvate in glucose metabolism, is inhibited in acetylation levels by heat stress, leading to reduced enzyme activity, blocked pyruvate oxidative decarboxylation, and impaired cellular energy metabolism (Zhou et al., 2025). Due to the inability of cells to efficiently convert pyruvate into acetyl-CoA for entry into the tricarboxylic acid cycle to produce energy, cells suffer from insufficient energy supply in high-temperature environments, exacerbating heatstroke-induced cell and tissue damage.

In the nucleus, histone acetylation levels also change due to heatstroke, affecting chromatin structure and gene transcriptional activity, influencing the expression of heat stress-related genes such as heat shock proteins, and participating in the body’s overall response to heat damage (Mundhara et al., 2021). Gene expression is a complex process executed by a group of transcription factors, which initiate divergent transcription from the core promoter regions of promoters and enhancers. Anniina et al. found that heat stress increases the acetylation levels of histones H3 and H4, loosening chromatin structure to promote transcription (Vihervaara et al., 2017). They also found that genes show distinct transcriptional directionality at core promoters, selectively assembling general transcription factors. A study in *Caenorhabditis elegans* found that histone deacetylase (HDAC) is necessary for activating the mitochondrial unfolded protein response during heat stress and induces the transcription of unfolded protein response-related gene ChIP by interacting with the genome organizer DVE-1, promoting innate immunity and extending lifespan (Shao et al., 2020). Meanwhile, the conserved evolutionary mechanism of HDAC1/2 in regulating mitochondrial homeostasis and longevity is presumably applicable to mammals. Additionally, acetylation of Hsp90 at K27 in *Aspergillus fumigatus* renders invasive aspergillus sensitive to heat stress; inhibiting Hsp90 deacetylation and increasing its acetylation levels can inhibit the growth of *Aspergillus fumigatus* and its resistance to azoles and echinocandins *in vitro* (Lamoth et al., 2014).

### 3.3 Changes and roles of ubiquitination modifications in heatstroke

Ubiquitination involves the covalent binding of ubiquitin molecules (a small protein consisting of 76 amino acids) to target proteins through the combined action of ubiquitin-activating enzymes, ubiquitin-conjugating enzymes, and ubiquitin ligases. Labeled target proteins are often recognized and degraded by the proteasome, regulating protein stability and levels, and playing important roles in numerous physiological processes such as cell cycle, immune response, and signal transduction (Damgaard, 2021).

Heat stress can induce ubiquitination of some damaged or misfolded proteins in cells, which are then degraded by the proteasome system to clear proteins that may adversely affect cellular functions and maintain the relative stability of the intracellular environment (Comyn et al., 2014). Nedd4 is one of the main E3 ligases inducing ubiquitination under heat stress. Fang et al. found that Nedd4 mainly targets cytoplasmic proteins under heat stress, and this ubiquitination process requires the involvement of the Hsp40 co-chaperone protein Ydj1, while specific binding motifs in substrates (PY Nedd4-binding motifs) also promote ubiquitination (Fang et al., 2014). The tumor suppressor p53 plays an important regulatory role in cellular stress responses, abnormal cell proliferation, and DNA damage. In normal cells, p53 is maintained at low expression levels through rapid degradation via the ubiquitin-dependent proteasome pathway (Wang et al., 2022). Under stimuli such as ionizing radiation and heat stress, p53 is activated as a transcription factor and induces the expression of genes such as p21, WAF1, gadd45, bax, p53AIP, and PUMA, thereby triggering cell cycle arrest and apoptosis (Mansky et al., 2023; Brooks and Gu, 2011). Wang et al. found that heat stress inhibits MDM2-mediated ubiquitination of p53, suppressing p53 degradation and leading to intracellular p53 accumulation to maintain stable p53 expression after heat stress (Wang and Chen, 2003).

Ubiquitination is crucial for the recovery of cellular activity after heatstroke. Eukaryotic cells respond to various cellular stresses by downregulating key cellular activities and sequestering cytoplasmic mRNA into structures called stress granules, accompanied by a global increase in ubiquitination (Shalgi et al., 2014; Zhang et al., 2018). Researchers from St. Jude Children’s Research Hospital used tandem ubiquitin binding entity (TUBE) proteomics—a method employing high-affinity ubiquitin-binding matrices to enrich and profile ubiquitinated substrates—to study ubiquitination changes in heat stress responses of *in vitro* cultured mammalian cells, detailing heatstroke-specific ubiquitination patterns (Maxwell et al., 2021). In human embryonic kidney 293T cells, they found that ubiquitinated proteins under heat stress are enriched in stress granules; when cellular activity recovers after heat stress, heat shock-induced ubiquitination is a prerequisite for p97/valosin-containing protein (VCP)-mediated stress granule disassembly and the restoration of normal cellular activities such as nucleocytoplasmic transport and protein translation (Maxwell et al., 2021).

However, in severe heatstroke with excessive heat damage, the ubiquitin-proteasome system may be overloaded or dysfunctional, leading to excessive ubiquitination and degradation of normally functioning proteins, thereby affecting normal cellular metabolism, signal transduction, and other physiological processes, and exacerbating organ function damage (Kuechler et al., 2021; Li et al., 2022). When cells are damaged by heat stress, HSP70 expression levels increase, and its ubiquitination levels also change (Nitika et al., 2020; Zhang et al., 2015). Under normal circumstances, HSP70 helps proteins fold correctly and prevents protein aggregation. However, when misfolded proteins increase, these proteins are ubiquitinated, and HSP70 interacts with these ubiquitinated proteins (Alagar Boopathy et al., 2022). On one hand, HSP70 clears heat-induced abnormal proteins through the ubiquitin-proteasome system to maintain intracellular stability; on the other hand, if ubiquitination is dysregulated, excessive ubiquitination leads to excessive degradation of HSP70 itself, reducing cellular protein folding and repair capabilities, making cells more vulnerable to heat damage and exacerbating the condition (Shi et al., 2011).

Additionally, In et al. found that heat stress promotes the E3 ubiquitin ligase RNF20/40 to act with its corresponding E2 ubiquitin-conjugating enzyme RAD6, monoubiquitinating lysine 381 of the heat shock transcription factor eEF1BδL and recruiting p-TEFb to the promoter region, thereby enhancing gene transcription (In et al., 2019). Furthermore, eEF1BδL and RNF20/40 interact with HSF1 to jointly promote the expression of heat shock genes (In et al., 2019).

### 3.4 Changes and roles of methylation modifications in heatstroke

Protein methylation refers to the transfer of methyl groups to specific amino acid residues (e.g., lysine, arginine) of proteins by methyltransferases (MTs). Based on differences in substrate amino acids, methyltransferases are classified into protein lysine methyltransferases (PKMTs) and protein arginine methyltransferases (PRMTs). Additionally, amino acids that can undergo methylation include histidine and aspartic acid. Methylation can change protein charge, structure, and interactions with other molecules, participating in gene expression regulation, cell differentiation, and other biological processes, with demethylation mechanisms also existing (Murn and Shi, 2017).

As nuclear proteins binding to DNA, post-translational modifications of histone N-terminal tails are key mechanisms regulating gene transcription and synergize with DNA methylation to participate in chromatin state regulation. Histone modifications play important roles in heat adaptation (AC), deacclimation (DeAC), and reacclimation (ReAC) related to heatstroke. Under moderate heat stimulation, the HSFA2 and HSFA3 complex in *Arabidopsis thaliana* promotes transcriptional memory by inducing high methylation of histone H3 lysine 4 (H3K4) to maintain body homeostasis (Friedrich et al., 2021). Additionally, histone release related to histone modifications can serve as a marker of heatstroke severity: serum histone levels in EHS dogs are significantly positively correlated with disease severity biomarkers (Bruchim et al., 2017a); histone H3 levels in plasma exosomes of EHS patients are closely related to organ dysfunction and disease severity [area under the curve (AUC) = 0.925 ] (Li et al., 2021).

### 3.5 Changes and roles of SUMOylation modifications in heatstroke

Small Ubiquitin-like Modifier (SUMO) modification, a core form of PTMs, is the covalent binding of three homologous proteins (SUMO1, SUMO2, SUMO3) to target proteins, regulating their subcellular localization, functions, and interaction networks (Wu and Huang, 2023). In heat stress responses, SUMO2/3 exhibit significant functional specificity, with only these two involved in heat stress responses, driving spatial rearrangement and functional remodeling of target proteins through the formation of poly-SUMO chains, providing key insights into the molecular pathological mechanisms of heatstroke (Liebelt et al., 2019).

Heatstroke, the terminal stage of extreme heat stress, can induce extensive remodeling of intracellular SUMOylation profiles. Yin et al. showed that high-temperature stimulation triggers SUMO2/3 modification of hundreds of proteins (e.g., 574 heat stress-responsive substrates identified by proteomics), involving core pathways such as cell cycle regulation, apoptotic signal transduction, protein folding/transport, and DNA damage repair (Fritah et al., 2014; Yin et al., 2012; Domingues et al., 2015; Golebiowski et al., 2009; Golebiowski et al., 2004; Gołebiowski et al., 2003). Brunet et al. found that SUMO2/3 modifies HSF1 (K298 site) and promotes its phosphorylation (S303/S307 sites), thereby enhancing heat shock gene transcriptional activity (Brunet Simioni et al., 2009). They further demonstrated that SUMO2/3 alleviates heat stress-induced genomic damage by modifying the DNA repair protein PARP1. SUMOylation of HSF1 at K298 represents a key regulatory node in heat shock gene transcription. Studies using SUMOylation-blocking mutations (K298R) have demonstrated abrogated HSF1 phosphorylation at S303/S307 and reduced transcriptional activity of heat shock genes, confirming the functional synergy between SUMOylation and phosphorylation (Brunet Simioni et al., 2009). Conversely, a phospho-mimicking mutation at S303/S307 (Ser→Asp) partially rescues the transcriptional defect caused by K298R, indicating hierarchical crosstalk between these modifications (Brunet Simioni et al., 2009). However, these mutations differ from *bona fide* SUMOylated/phosphorylated HSF1: native modifications are reversible and spatially restricted, whereas mutations induce constitutive changes that may disrupt context-dependent regulation. Liu et al. confirmed through animal models that the incidence of heat convulsions in SUMO-overexpressing mice is significantly lower than in wild-type mice, with prolonged convulsion latency and shortened duration (Zhang et al., 2017; Liu et al., 2017) (Table 2).

**TABLE 2:** Relevant PTM sites on major HSPs in heat stress-related diseases.

HSP/Regulator	PTM type	Specific Site(s)	Regulatory mechanism & function in heat stress	References
HSP27	Phosphorylation	Ser135, Arg136	Phosphorylated by p38 MAPK/MK2; recruits to stressed lysosomes, interacts with p62, promotes lysosomal autophagy	Gallagher et al. (2024)
	Phosphorylation	Ser82, Ser78	Phosphorylated by MK2; promotes stress granule assembly and clearance of damaged organelles	Gallagher et al. (2024)
HSP70	Ubiquitination	Multiple sites	Ubiquitinated by CHIP; clears heat-induced misfolded proteins via UPS; excessive ubiquitination reduces its chaperone activity	Brooks and Gu (2011), Kuechler et al. (2021)
HSP90	Acetylation	K27 (in Aspergillus fumigatus)	Inhibits growth and drug resistance under heat stress; potential conservation in mammalian HSP90	Mundhara et al. (2021)
HSF1	Phosphorylation	Ser303, Ser307	Mediated by p38 MAPK; promotes trimerization, nuclear translocation, and transcriptional activation of HSP genes	Brunet Simioni et al. (2009)
	SUMOylation	K298	Enhances phosphorylation at Ser303/307; synergistically amplifies heat shock gene transcription	Brunet Simioni et al. (2009)

### 3.6 Changes and roles of S-Nitrosylation modifications in heatstroke

S-nitrosylation is a redox-sensitive reversible PTM in which NO or its derivatives react with cysteine thiols (-SH) of substrate proteins to form S-nitrosothiols (SNO) (Benhar et al., 2009). Under physiological conditions, S-nitrosylation regulates protein functions by affecting protein structure, stability, subcellular localization, transcriptional activity, and intermolecular interactions (Haldar and Stamler, 2013; Tang et al., 2023). S-nitrosylation is involved in the occurrence and development of various liver diseases, and its role must be discussed for specific substrates in specific environments (Cui et al., 2024; Kim et al., 2000).

High temperatures promote the generation of superoxide anions (O_2_^−^) by activating NADPH oxidase (NOX) and inducing mitochondrial dysfunction (Kellogg et al., 1985; Hall et al., 2001). O_2_^−^ reacts with NO to form peroxynitrite (ONOO^−^), exacerbating nitrosative stress and driving S-nitrosylation (Foster and Stamler, 2004). Meanwhile, heat stress induces the release of various inflammatory mediators such as TNF-α and IL-6, stimulating the expression and activity of nitric oxide synthase (NOS) (Alzeer et al., 1999). NOS catalyzes the production of NO from L-arginine, increasing local intracellular NO concentration (Hall et al., 2001; Alzeer et al., 1999; Epstein and Yanovich, 2019), which then reacts with protein cysteine residues to form SNO. The activity and function of some intracellular redox enzymes are also affected and altered, such as reduced content and activity of glutathione (GSH) and its related enzymes (glutathione peroxidase (GSH-Px), glutathione reductase (GR), and S-nitrosoglutathione reductase (GSNOR)) in the important intracellular antioxidant defense system (Song et al., 2022). This disrupts intracellular redox balance, altering the oxidation state of sulfur-containing compounds in cells, which is conducive to the formation of S-nitrosoglutathione (GSNO) (Hess and Stamler, 2012). GSNO, an efficient NO donor, transfers SNO groups to protein cysteine residues through transnitrosylation. Additionally, under oxidative stress and metabolic disorders caused by heatstroke, intracellular pH changes affect protein charge distribution and conformation, making proteins containing cysteine residues more likely to expose their reactive sites, thereby increasing reactivity with NO or other enzymatic reagents (Bruchim et al., 2017b). These factors provide an environmental basis for S-nitrosylation induced by heat stress.

Research on protein S-nitrosylation in heatstroke is relatively limited. William et al. showed that the RyR1 Y522S mutation leads to sarcoplasmic reticulum Ca^2+^ leakage, increasing cytoplasmic Ca^2+^ and activating NOS, promoting the production of reactive nitrogen species (RNS), and thereby inducing S-nitrosylation of RyR1 (Durham et al., 2008). This modification significantly increases the temperature sensitivity of the mutant RyR1 channel, making it more prone to opening at high temperatures, further exacerbating Ca^2+^ leakage, and forming a positive feedback loop of “Ca^2+^ leakage→RNS↑→S-nitrosylation→exacerbated Ca^2+^ leakage.” This loop causes persistent muscle rigidity, rhabdomyolysis, and mitochondrial damage (swelling, lipid peroxidation), ultimately leading to multiple organ failure and sudden death (Durham et al., 2008). The antioxidant N-acetylcysteine (NAC) can block this process and alleviate heatstroke phenotypes in mice, highlighting the important role of protein S-nitrosylation in heatstroke (Durham et al., 2008).

### 3.7 PTM crosstalk in heat stress

**FIGURE 1:**
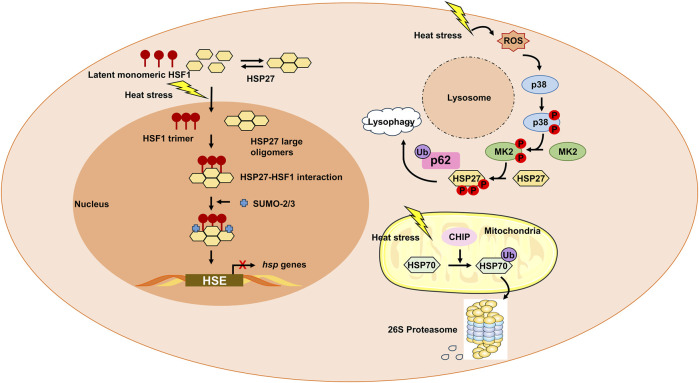
Post-translational modifications (PTMs) of heat shock proteins (HSPs) and their functional roles in heat stress responses. Heat stress triggers dynamic PTMs of HSPs and their regulators, orchestrating cytoprotective and pathogenic outcomes. Catalyzed by SUMOylases, SUMO2/3 modification of HSF1 enhances its phosphorylation at Ser303/307 (via p38 MAPK) (Brunet Simioni et al., 2009), promoting trimerization, nuclear translocation, and binding to heat shock elements (HSEs) on HSP gene promoters. Phospho-mimicking mutations rescue transcriptional activity, while SUMO-blocking mutations (K298R) abrogate this synergy, highlighting PTM crosstalk. Activated p38 MAPK/MK2 phosphorylates HSP27, driving its recruitment to stressed lysosomes (Gallagher et al., 2024). Phosphorylated HSP27 interacts with ubiquitinated p62, promoting lysosomal autophagy to clear damaged organelles. MK2-mediated phosphorylation induces HSP27 oligomer disassembly, facilitating stress granule assembly and clearance of misfolded proteins. CHIP (E3 ligase) ubiquitinates HSP70, targeting misfolded proteins for proteasomal degradation (Brooks and Gu, 2011; Kuechler et al., 2021). Excessive ubiquitination reduces HSP70 chaperone activity, exacerbating protein aggregation.

PTM crosstalk plays a critical role in integrating heat stress signals, as exemplified by several key interactions. SUMOylation of HSF1 at K298 enhances its phosphorylation at Ser303/307, synergistically amplifying heat shock gene transcription (Brunet Simioni et al., 2009); p53 stability is bidirectionally regulated by phosphorylation (which promotes activation) and MDM2-mediated ubiquitination (which drives degradation), collectively determining cell fate under heat stress (Wang and Chen, 2003); and phosphorylation of HSP27 facilitates its interaction with ubiquitinated p62, linking autophagy to stress granule clearance (Gallagher et al., 2024). Notably, PTMs exhibit distinct tissue and stage specificity: early heat stress is dominated by protective PTMs such as HSP27 phosphorylation (which promotes autophagy), whereas severe heatstroke is characterized by pathogenic PTMs, including S-nitrosylation of RyR1 in muscle and hypophosphorylation of AMPK in the heart (Durham et al., 2008; Tian et al., 2023). Tissue-specific PTM patterns further emerge, with histone modifications (e.g., H3K4me3) driving transcriptional memory in the brain and S-nitrosylation dominating redox imbalance in the liver (Li et al., 2021; Foster and Stamler, 2004). However, unresolved questions remain, such as the divergent regulation of NF-κB (activation in endothelium vs inhibition in osteosarcoma cells under high temperature), which may stem from cell-type-specific PTM crosstalk (Liu et al., 2015; Kardyńska et al., 2018), and the unclear relationship (mutually exclusive or cooperative) between HSP27 phosphorylation and SUMOylation. These PTM crosstalk mechanisms—such as the enhancement of HSF1 phosphorylation by SUMOylation and the coordination of HSP27 phosphorylation with p62 ubiquitination—are visually summarized in the figure illustrating post-translational modifications of heat shock proteins and their functional roles in heat stress responses (Figure 1).

## 4 PTMs as biomarkers and therapeutic targets for heat stress-related diseases

### 4.1 PTMs as biomarkers for diagnosis and prognosis

The dynamic landscape of PTMs in heat stress-related diseases presents dual translational value: as precision biomarkers for early diagnosis and as therapeutic targets for intervention. Specific PTM alterations exhibit strong correlations with disease severity, enabling prognostic stratification beyond traditional biomarkers (Maxwell et al., 2021; Li et al., 2021). For instance, elevated histone H3 levels in plasma exosomes (AUC = 0.925) sensitively reflect multi-organ dysfunction in EHS patients (Li et al., 2021), while heat stress-induced ubiquitination profiles provide molecular fingerprints for early warning (Maxwell et al., 2021). These findings position PTM-based detection methods—such as phosphorylation site quantification and SUMOylation substrate mapping—as promising tools for clinical risk assessment.

### 4.2 PTMs as therapeutic targets: Interventions and challenges

Therapeutic strategies targeting PTM networks show significant potential but face translational challenges. Experimental interventions include.

- S-Nitrosylation inhibition via N-acetylcysteine (NAC), which disrupts RyR1-mediated Ca^2+^ leakage and reduces mortality in preclinical models (Durham et al., 2008).
- Phosphorylation restoration using metformin to activate AMPK Thr172 phosphorylation, reversing cardiac metabolic dysfunction (Tian et al., 2023).
- Epigenetic modulation with HDAC inhibitors (e.g., vorinostat) to enhance HSP70 expression and proteostasis (Shao et al., 2020).
- SUMOylation/ubiquitination modulation through small molecules targeting HSF1 activation or p53-MDM2 regulation (Brunet Simioni et al., 2009; Brooks and Gu, 2011).

However, these approaches confront challenges of specificity (e.g., p38 MAPK inhibitors disrupting both protective and pathogenic pathways), tissue delivery limitations (requiring nanocarriers for organ-targeted therapy), off-target effects (e.g., HDAC inhibitors altering epigenetic memory), and validation hurdles (necessitating large cohort studies for PTM-based biomarkers).

### 4.3 Integration of mutant studies and bona fide PTM analyses

Critical insights into PTM mechanisms derive from studies employing PTM-mimicking mutants and PTM-deficient variants. While such mutants establish site-specific roles, they cannot replicate the dynamic, enzyme-regulated nature of native PTMs—where kinase/phosphatase cycles or SUMOylation/deSUMOylation balances adapt to physiological cues. Thus, integrating mutant data with analyses of *bona fide* modified proteins (e.g., S-nitrosylated RyR1 (Durham et al., 2008) or ubiquitinated stress granules (Maxwell et al., 2021)) provides a comprehensive understanding of PTM functionality in heat stress pathologies.

### 4.4 Organ-specific PTM patterns in therapeutic translation

Organ-specific PTM patterns play distinct roles in mediating heat stress-induced pathophysiology across vulnerable tissues. In the heart, heatstroke inhibits AMPK phosphorylation at Thr172, shifting myocardial metabolism toward glycolysis, which leads to lactic acid accumulation and cardiac dysfunction (Tian et al., 2023; Laitano et al., 2020; Zheng et al., 1985; Roths et al., 2023); conversely, phosphorylation of HSP27 at Ser135 in cardiomyocytes confers protection against lysosomal damage (Gallagher et al., 2024). In the brain, heat stress enhances acetylation of histones H3 and H4, which loosens chromatin structure to promote transcription of neuroprotective genes (Li et al., 2021), while plasma exosomal levels of histone H3 correlate with the severity of cerebral edema, serving as a potential prognostic marker. In the liver, S-nitrosylation of antioxidant enzymes (e.g., glutathione peroxidase) impairs redox homeostasis, exacerbating hepatic inflammatory responses (Foster and Stamler, 2004); additionally, ubiquitination of hepatic stress granule proteins is critical for restoring cellular function during post-heat recovery (Maxwell et al., 2021) (Table 3).

**TABLE 3:** Comparative summary of PTMs in heat stress-related diseases.

PTM type	Modifying enzymes	Target proteins	Biological effects	Tissue involvement	Clinical relevance	Potential in drug development	References
Phosphorylation	p38 MAPK, MK2, ERK, AMPK	HSP27 (Ser135), NF-κB p65, AMPK (Thr172)	Regulates autophagy (HSP27), inflammation (NF-κB), metabolism (AMPK)	Heart, endothelium	AMPK activators as potential cardioprotectants; p-p65 as inflammation marker	Kinase activators (e.g., metformin for AMPK) or inhibitors (e.g., p38 MAPK inhibitors)	Gallagher et al. (2024), Tian et al. (2023), Wang et al. (2024)
Acetylation	HDACs, acetyltransferases	Histones (H3/H4), PDH	Alters transcription (histones), inhibits energy metabolism (PDH)	Brain, liver	HDAC inhibitors to enhance HSP expression; acetyl-PDH as metabolic marker	HDAC inhibitors (e.g., vorinostat) to enhance HSP expression	Shao et al. (2020), Xiong and Guan. (2012), In et al. (2019)
Ubiquitination	Nedd4, MDM2, RNF20/40	Misfolded proteins, p53, eEF1BδL	Clears damaged proteins (UPS), regulates p53 stability, enhances HSP transcription	Ubiquitous, stress granules	Ubiquitin profiles as early warning markers; p53 stabilizers in therapy	E3 ligase modulators (e.g., targeting MDM2 for p53 stabilization)	Maxwell et al. (2021), Wang and Chen. (2003), Lamoth et al. (2014), Kuechler et al. (2021)
SUMOylation	Ubc9, SUMOylases	HSF1 (K298), PARP1	Amplifies heat shock response (HSF1), repairs DNA (PARP1)	Ubiquitous	SUMO2/3 modulators to reduce heat convulsions	SUMOylases/deSUMOylases modulators to mitigate heat-induced genomic damage	Brunet Simioni et al. (2009), Fritah et al. (2014), Yin et al. (2012)
S-nitrosylation	NOS, GSNOR	RyR1, GSH-Px	Triggers Ca^2+^leakage (RyR1), impairs antioxidant defense (GSH-Px)	Skeletal muscle, liver	NAC as inhibitor (clinical use in animal models); SNO-RyR1 as severity marker	NAC (blocks S-nitrosylation) for reducing heatstroke mortality	Durham et al. (2008), Zhang et al. (2017), Haldar and Stamler. (2013)

## 5 Conclusion and outlook

This article comprehensively elaborates on the core roles of PTMs such as phosphorylation, acetylation, ubiquitination, methylation, SUMOylation, and S-nitrosylation in heat stress-related diseases. Studies have shown that heat stress dynamically reshapes the functional states of key proteins (e.g., HSF1, NF-κB, p53) by affecting modification enzyme networks such as kinases/phosphatases and acetyltransferases/deacetylases, thereby regulating pathophysiological processes including cellular stress responses, metabolic adaptation, inflammatory storms, and programmed death (Wang et al., 2024; Liu et al., 2015; Lamoth et al., 2014). These PTMs are not only molecular hubs linking high-temperature stimulation and multi-organ damage but also their temporal changes in modification profiles can serve as “molecular rulers” for evaluating disease progression.

Current research still has significant limitations. Firstly, most mechanisms are based on cell or animal models, lacking dynamic PTM profiles at the tissue level in heatstroke patients. Secondly, the crosstalk between different PTMs has not been analyzed in the context of heat stress. Thirdly, research on organ-specific modifications, such as differential regulatory networks in brain, liver, and heart damage, is insufficient. Future studies should also focus on mutational analyses of key PTM sites (e.g., HSP27 Ser135) to clarify their causal roles in heat stress responses. And should focus on combining modification omics with single-cell sequencing technology to map spatiotemporal modification profiles at different stages of heat stress; develop *in vivo in situ* PTM imaging methods to track the causal relationship between modification changes and organ damage; design tissue-specific nanocarriers to precisely regulate PTM enzyme activity in diseased organs; and further explore the application value of PTM biomarkers such as exosomal histone modifications in early diagnosis and stratified treatment of heatstroke. Through interdisciplinary research, PTM regulatory networks are expected to become a key breakthrough in deciphering the pathological code of heat stress diseases, providing new targets for prevention and treatment strategies.
