# Correction: Post-translational modifications in heat stress-related diseases

**DOI:** 10.3389/fmolb.2025.1723915

**Published:** 2025-11-07

**Authors:** Qi Cui, Keran Jia, Fang Li, Jie Zheng, Fukun Wang

**Affiliations:** 1 Clinical Laboratory, Bethune International Peace Hospital, Shijiazhuang, Hebei, China; 2 Nursing Department, Bethune International Peace Hospital, Shijiazhuang, Hebei, China

**Keywords:** heat stress, post-translational modifications, phosphorylation, acetylation, ubiquitination, methylation, S-nitrosylation

Author Qi Cui was erroneously assigned to affiliation Postdoctoral Mobile Station for Clinical Medicine, Hebei Medical University, Shijiazhuang, Hebei, China. This affiliation has now been removed for author Qi Cui.

The original article has been updated.

